# A Longitudinal Study of Changes in Thyroid Related Hormones among Pregnant Women Residing in an Iodine Deficient Urban Area

**DOI:** 10.1155/2013/234031

**Published:** 2013-10-21

**Authors:** Shan Elahi, Zaib Hussain

**Affiliations:** ^1^Centre for Nuclear Medicine (CENUM), P.O. Box 53, Mayo Hospital, Lahore, Pakistan; ^2^Institute of Chemistry, New Campus, University of the Punjab, Lahore, Pakistan

## Abstract

*Problem Statement*. Thyroid gland in women undergoes functional changes during pregnancy. A few studies have described such changes in pregnant women residing in iodine deficient areas. *Objective*. To document these changes in pregnant women residing in Lahore, a low iodine intake urban area of Pakistan. *Patients and Methods*. In 254 pregnant women, data of FT_4_, FT_3_, and TSH during the first and subsequent trimesters were obtained and compared with those of 110 nonpregnant women. These hormones were determined in serum by radioimmunoassay (RIA) techniques using commercial kits. *Results*. Compared to nonpregnant women mean FT_4_ level was decreased, and FT_3_ and TSH increased significantly (*P* < 0.05) in pregnant women. A negative correlation of FT_4_ with TSH was observed in all three trimesters. Serum FT_3_ was positively correlated with TSH only during the third trimester. As a function of gestation time, FT_4_ levels progressively decreased, and FT_3_ and TSH levels increased significantly (one-way ANOVA *F* = 108.2, 17.3, and 44.8, resp.; all *P* < 0.05) exhibiting thyroid gland adaptations. *Conclusion*. Pregnancy is associated with significant alterations in thyroid function due to low iodine intake in women residing in study area. The compensated thyroid function poses a risk of thyroid failure in a number of pregnant women.

## 1. Introduction

Over the past twenty years, there has been a major expansion of our knowledge regarding the relationships between pregnancy and the thyroid hormones. The most important finding in this regard is that maternal thyroid hormones play a vital role in early fetal brain formation, and their deficiency may impair future neuropsychological development of the fetus [1–3]. Pregnancy is associated with certain physiological changes for which the maternal thyroid gland has to adapt accordingly [1, 4]. The first factor is the adjustment of bound to free ratio of T_4_ and T_3_ against the marked increase in the circulating levels of thyroxin binding globulin (TBG) levels due to enhanced estrogen production. The second factor is the direct stimulation of the thyroid gland by elevated concentration of human chorionic gonadotropin (hCG). These two factors occur in the first trimester of pregnancy [1]. The third factor is the increased enzymatic activity of type III monodeiodinase. It converts T_4_ to reverse T_3_ (rT_3_) and thus increases the turnover rate of maternal T_4_ at the placental level. It is operative in later stages of pregnancy [1, 4].

The above mentioned thyroid stimulants during pregnancy enhance maternal iodine requirement which is further exacerbated due to increased renal clearance of iodine from the kidney. Moreover, a part of the available iodine from the maternal circulation is diverted to fetal thyroid gland which becomes progressively functional by the end of the first trimester [1, 4]. Thus, the regulation of maternal thyroid function is complex and varies with each stage of pregnancy [1]. In case of pregnant women taking sufficient iodine, the thyroid gland readily adjusts the hormonal output and achieves the new equilibrium state. This equilibrium is difficult to achieve when iodine intake of pregnant women is low [1, 4, 5]. In this situation, maternal thyroid function tends to adjust itself and these adjustments may range from physiological adaptation to pathological derangement depending on the degree of iodine deficiency [1, 6]. However, the longitudinal studies addressing thyroid function during pregnancy in mild to moderate iodine deficient populations are not fully elucidated [7]. 

Pakistan is a developing country. Typical Pakistani diet is reported to contain 40 *μ*g/day iodine content that is 3.8 times lower than 150 *μ*g/day,recommended for adult subjects [8]. The existence of mild to moderate iodine deficiency has been reported in women of childbearing age residing in Lahore, the second largest city of Pakistan [9]. Recently as a part of a longitudinal study in pregnant women, we have described the detail of iodine deficiency in pregnant as well as nonpregnant women residing in this city [10, 11]. In the present paper, the data of sera FT_4_, FT_3_, and TSH of the same cohort of pregnant women during successive trimesters is compared with those of nonpregnant women. Also, the mean levels of thyroid related hormone associated with each trimester are compared mutually to elucidate their trends of significant changes (if any) during pregnancy. 

## 2. Patients and Methods

The field work of this study was carried out from March 2002 to September 2005, and laboratory work was completed up to 2007. All participant women resided in the municipal jurisdiction of Lahore and belonged to the middle socio-economic strata. These women attended antenatal clinics at the outpatient department of obstetrics and gynecology, Government Mian Munshi Hospital. At the first presentation, all of them underwent an ultrasound scan in order to confirm the pregnancy. Ultrasonography was performed inside the hospital using standard procedure on an ultrasound machine (Siemens, Germany, Model Solo line SL-1, 7.5 MHz) with linear transducer. Those pregnant women with gestational age within the first trimester were enrolled for this longitudinal study. The objective of the study was explained to each woman and a written consent of participation was obtained. A detailed history of past gynecological events of each patient was noted on a performa devised for this study. Each woman was examined for the presence of goiter and inquired of iodine salt intake and past history of thyroid diseases. Women with hepatic, renal, or evident endocrine disorders, history of immunosuppressive therapy, history of thyroid surgery or thyroid dysfunction, or on any form of drug treatment were excluded from the study. 

At first presentation, the mean gestational age of the study sample was 9.9 ± 2.4 weeks. From each selected woman a 5 mL blood sample was collected for thyroid related hormones (FT_4_, FT_3_, and TSH). These women were advised to attend the antenatal clinic after every two weeks. Further blood sample was drawn from each woman in the mid of the second trimester (17–25 weeks; mean: 21.6 ± 2.6) and the third trimester (26–35 weeks; mean: 30.6 ± 1.8). Nonpregnant women were randomly selected as a reference population for comparative purposes (control). They were healthy, nonlactating for the previous two years, and free of any thyroid illness and had regular menstrual cycles. These women were not using any oral drugs that could interfere with serum thyroid hormone and TSH levels. A 5 mL blood sample was collected from each control woman. The Institutional Review Board at the University of the Punjab, Lahore, approved the study. 

The maternal blood samples were allowed to coagulate at room temperature, and sera were separated by centrifugation (2000 ×g) for 5 minutes with a centrifuge (Model NF 1215, Nuve, Turkey). Collected sera were divided into multiple aliquots and stored at minus 80°C until analysis. Measurement of FT_4_, FT_3_, and TSH was carried out at RIA laboratories, Centre for Nuclear Medicine (CENUM), Mayo Hospital, Lahore, by using commercial kits of Immunotech Inc. (Beckman Coulter, Czech Republic and France). Sera FT_3_ and FT_4_ were determined by radioimmunoassay (RIA), and TSH was estimated by immunoradiometric assay (IRMA) using a computerized gamma counter (Cap-RIA 16, Capintec Inc., PA, USA). Average intera-ssay and intraassay precisions respectively, as indicated by the percentage of coefficients of variation (%CV) were FT_4_ (3.8% and 4.9%), FT_3_ (3.3% and 7.8%), and TSH (4.4% and 5.9%). 

Data were analyzed using the statistical package for social sciences (SPSS) version 13.0 for Windows (SPSS Inc. 2003, MapInfo Corp., New York, USA). The FT_4_ and FT_3_ data were normally distributed. The TSH data were not normally distributed and hence log transformed. Results were presented as means (±SD) or otherwise specified. Correlations were carried out using regression analysis. Mean levels of FT_4_, FT_3_, and TSH in pregnant and nonpregnant women were compared by Student's *t*-test. In pregnant women repeated measures one-way analysis of variance (ANOVA) and the Bonferroni test were used to determine the trend of changes in FT_4_, FT_3_ and TSH levels across different trimesters of pregnancy. All results were considered statistically significant at *P* < 0.05.

## 3. Results

A total of 310 women were examined but only 280 women fulfilled the criteria for selection and volunteered to participate in this study. During the course of the study, 26 women either refused or dropped out of the study due to other reasons. Thus, the study sample consisted of 254 women. A total of 110 nonpregnant women were studied as controls. The relevant characteristics of both groups of women are presented in Table 1. Both groups were comparable with respect to age, parity, and current use of iodine salt. The cohort of pregnant women comprised 84 (33.1%) primigravidae, and the remaining 170 women have had 1–9 pregnancies (average, 4.2) and from 0 to 7 alive children (average, 2.4). Forty-three percent of these women had experienced miscarriage or abortion during their previous pregnancies. 

As compared to nonpregnant normal ranges, the percentages of pregnant women falling in the category of low or high value are shown in Table 2. About two-thirds of the pregnant women had all their thyroid function tests within nonpregnant normal range throughout the pregnancy. Overall 15–25% of pregnant women had their FT_4_ and TSH near the lower limits of the normal ranges, while less than 10% of women had supranormal FT_3_ and TSH during the entire course of gestation. More than 20% of pregnant women had lower FT_4_ and only 2% had higher TSH throughout pregnancy. The smallest decrease and highest increase were noted in FT_3_ levels. 

Pearson's correlation coefficient (*r*) between TSH and thyroid hormone (FT_4_ and FT_3_) in nonpregnant and pregnant women (each trimester) is given in Table 3. Unlike nonpregnant women, a definite correlation was observed between thyroid hormone and TSH in pregnant women. Serum FT_4_ was negatively correlated with serum TSH in all three trimesters, and the magnitude of this correlation increased with the progression of pregnancy. Serum FT_3_ was correlated negatively with TSH during the first trimester. However, it was positively correlated with TSH during the third trimester of pregnancy. 

The mean levels of thyroid related hormones in pregnant women (during successive trimesters) are shown in Table 4. Compared to nonpregnant women, FT_4_ levels in pregnant women significantly (*P* < 0.05) decreased by 17.9% in the first trimester, 28.2% in the second trimester, and 26.9% in the third trimester. The respective numbers of pregnant women falling below the lower limit of nonpregnant normal range were 53 (20.9%), 67 (26.4%), and 60 (23.6%). As a function of gestation time, overall FT_4_ levels decreased significantly (one-way ANOVA *F* = 108.2; *P* < 0.05) in pregnant women. Compared to the first trimester, FT_4_ levels decreased significantly in the second and the third trimesters (both *P* < 0.05). However, there was no significant difference between the second and the third trimesters (*P* = 0.485). 

Serum FT_3_ levels increased in the first trimester by 18.7% (*P* < 0.05), in the second trimester by 12.5% (*P* < 0.05), and in the third trimester by 21.8% (*P* < 0.05) as compared to nonpregnant women. The number of pregnant women with FT_3_ concentration above the upper limit of nonpregnant normal range in the first, the second, and the third trimesters was 18 (7%), 13 (5%), and 22 (9%), respectively. The overall increase in serum FT_3_ levels was significant (one-way ANOVA *F* = 17.3; *P* < 0.05). However, intertrimester comparison revealed that unlike FT_4_ and TSH, changes in FT_3_ levels were trimester specific. Compared to the first trimester, serum FT_3_ levels significantly decreased during the second trimester but increased from the second trimester to the third trimester (both *P* < 0.05). However, the difference between the first and the third trimesters in FT_3_ levels was not significant (*P* = 0.55). 

The changes in serum TSH levels were diametrically opposite to that of FT_4_. Overall serum TSH was increased significantly (one-way ANOVA *F* = 44.8; *P* < 0.05) during the entire course of pregnancy. Serum TSH levels decreased significantly (*P* = 0.05) during the first trimester by 20% but increased significantly (*P* < 0.05) during the second trimester by 26.7% and the third trimester by 60% (*P* < 0.05) as compared to nonpregnant women. During the first trimester 37 (14.6%) women had suppressed TSH levels. Compared to values on the first trimester, TSH levels increased significantly (*P* < 0.05) during the second as well as the third trimester. Increase in serum TSH from the second trimester to the third trimester was also significant (*P* < 0.05). During the first, the second, and the third trimester, the number of women with TSH levels above the upper limit of nonpregnant normal range was 5 (2.0%), 6 (2.4%), and 14 (5.5%), respectively. Analysis of concomitant changes in sera TSH and FT_4_ levels revealed that the relation between sera FT_4_ and TSH levels was not essentially antagonistic during pregnancy. In the first trimester, FT_4_ concentrations in all women with suppressed TSH were not above the upper limit of the normal range. Conversely, in the third trimester all women with raised serum TSH had FT_4_ levels below lower limit of nonpregnant normal range (transient hypothyroidism). 

## 4. Discussion

This study was planned to document the gestational associated changes in thyroid related hormones with respect to nonpregnant women residing in the same area. Both groups of women, as described earlier [10, 11], were mild to moderate iodine deficient, and about 30% of them were taking iodized salt (Table 1). The present results showed that, as compared to nonpregnant state, the correlation between TSH and thyroid hormones was significantly (*P* < 0.05) altered in pregnancy (Table 3). The thyroidal adjustments in pregnancy occurred in two phases: (a) compared to nonpregnant state there was a decrease in FT_4_, FT_3_, and TSH in early pregnancy and (b) considering early pregnancy FT_4_, FT_3_, and TSH levels as baseline, serum FT_4_ continued to decrease with parallel increase in FT_3_ and TSH levels in subsequent trimesters. The gradual decline in FT_4_ with the progression of pregnancy is consistent with previous longitudinal [12, 13] as well as cross-sectional studies [14–17]. In the present study, serum FT_4_ significantly decreased by more than 26% during the second and the third trimester as compared to the lower limit of normal value in nonpregnant women. Berghout et al. (1994) in a review article have reported this figure up to 30% in pregnant women residing in both iodine deficient and sufficient areas [18]. In pregnant women, an intertrimester comparison showed that FT_4_ concentration was relatively high during the first trimester which continued to decrease in subsequent trimesters (Table 4). This observation is in accordance with the above mentioned studies [12–17]. The reason proposed for these relatively elevated FT_4_ levels during early pregnancy is the extremely high concentrations of hCG at this stage that stimulates thyroid hormone secretion [1, 6]. An interesting but still unproved reason is the presence of the fetus itself requiring more FT_4_ for brain development during early pregnancy [3]. This speculation is supported by a study conducted in multifetal pregnancies where increase in FT_4_ concentration was significantly related to the number of fetuses [19]. In spite of the relatively higher FT_4_ concentration during the first trimester, 22 (8.7% of total sample) women had critically low FT_4_ concentration with concordant normal TSH levels. This condition is referred to as maternal hypothyroxinemia and is common in pregnant women residing in iodine deficient areas. Recent studies had proved this condition to be fatal for fetus brain development [2, 20].

Our results showed that compared to baseline value, FT_3_ levels were lower during the second trimester in pregnant women.This observation is in accordance to studies carried out in iodine sufficient areas [18] as well as in countries where iodine supplementation is recently introduced [14, 16, 17]. However, in these studies a decrease in FT_3_ levels was observed throughout the progression of pregnancy. The reason proposed for this phenomenon is the physiological adaptation enabling energy conservation due to high metabolic demands of pregnancy [18, 21]. This seems plausible as the decrease in FT_3_ levels is significantly correlated with the increase in BMR in late pregnancy [22]. In fact, the downregulation of the thyroid hormone action as indicated by the decrease in FT_4_ and FT_3_ contributes to the saving of energy during pregnancy [21, 22]. Another proposed reason is the increased production of reverse-T_3_ during pregnancy which like nonthyroidal illness decreases the synthesis of T_3_ [18]. However, our results are in contrast with this observation, and we have noted an increase in FT_3_ concentration from the second to the third trimester. Glinoer et al. (1990) in Belgium [4] and Sánchez-Vega et al. (2008) in Spain [12] had already noted the same observation. However, Kumar et al. (2003) in India observed an increase in FT_3_ only in the first and the second trimesters [13]. This discrepancy may be due to the difference in degree of iodine deficiency in pregnant women residing in different geographical locations. When iodine is insufficient, the maternal thyroid responds immediately with auto regulatory mechanisms, among which are the increased synthesis and secretion of T_3_ at the expense of T_4_. Consequently, maternal circulating total T_4_ and FT_4_ decrease, but total T_3_ and FT_3_ remain the same or even increase, thus avoiding an increase in circulating TSH [4, 12]. Another reason may be the diversion of a part of maternal iodine to fetal thyroid gland during the second half of pregnancy that consequently shifts the maternal thyroid gland to prefer rational synthesis of FT_3_ instead of FT_4_ to maintain euthyroidism during this period of relative iodine deficiency. 

Compared to nonpregnant women, the relatively low TSH in pregnant women during the first trimester was due to TSH suppression in 14% of them. This early pregnancy TSH suppression is attributed to extremely high concentration of hCG that has TSH-like activity [23] and inhibits thyrotropin-releasing hormone (TRH) secretion [24]. It is plausible as both TSH and hCG are heterodimeric glycoproteins composed of a common *α*-subunit, and they share considerable similarity in their ß-subunits with similar receptors [1]. This additional stimulation of thyroid gland diminishes during the second and the third trimesters [21, 23]. The increase in TSH levels during pregnancy is reported in many studies [12–16, 25]. We also observed an overall increase in serum TSH levels that was significant only during the second and the third trimesters. Panesar et al. (2001) and Ardawi et al. (2002) had observed an increase in TSH only during the third trimester [14, 21]. The difference in population iodine intakes seems to be the decisive factor in this regard. 

Thus, pregnancy in our mild to moderate iodine deficient women was associated with significant alterations in thyroid related hormones. Due to the limited supply of iodine, a progressive decrease in the thyroid function of pregnant women was observed. There was an overall decrease in FT_4_ and an increase in FT_3_ and TSH in pregnant women from the first to the third trimester. Thyroid glands in pregnant women appeared to compensate the decrease in FT_4_ levels by increasing FT_3_ synthesis as well as steadily increasing TSH concentration that kept on enhancing thyroid gland stimulation. Thus, compared to nonpregnant state, a tendency toward compensated thyroid function during pregnancy was observed that may lead to thyroid failure. This risk may be more aggravated in case of repeated pregnancies. This study warrants the need for establishment of gestation-specific reference intervals for thyroid related hormone to describe thyroid hormone deficiency in pregnant women [7, 26]. 

## Figures and Tables

**Table 1:** Characteristics of pregnant and control women*.

Group	Number	Age (year)	Parity	Number (%) of women taking iodized salt
Pregnant	254	24.2 ± 4.9^a^	3.5 ± 2.3^a^	87 (34.2)^a^
Nonpregnant	110	26.7 ± 4.8^a^	3.7 ± 2.3^a^	31 (28.2)^a^

*Similar superscript letters indicate statistically nonsignificant difference in the mean values of the two groups.

**Table 2:** Distribution of lower or higher thyroid function tests in pregnant women as compared to the normal ranges of nonpregnant women.

Thyroid hormone	1st trimester *n* (%)	2nd trimester *n* (%)	3rd trimester *n* (%)
FT_4_ (11.0–22.0 pmol/L)*			
Low value	53 (20.9)	67 (26.4)	60 (23.6)
High value	5 (2.0)	0	0
FT_3_ (2.5–5.8 pmol/L)			
Low value	7 (2.8)	0	0
High value	18 (7.1)	13 (5.1)	22 (8.7)
TSH (0.3–4.0 mIU/L)			
Low value	37 (14.6)	7 (2.8)	0
High value	5 (2.0)	6 (2.4)	14 (5.5)

*Normal range derived as 95% confidence interval.

**Table 3:** Correlation coefficients (Pearson) between TSH and thyroid hormones in nonpregnant and pregnant women.

	Nonpregnant women	Pregnant women		
		1st trimester	2nd trimester	3rd trimester
TSH and FT_4_	−0.055^NS^	−0.206*	−0.304*	−0.460*
TSH and FT_3_	0.129^NS^	−0.328*	0.069^NS^	0.206*

*Significant at *P* < 0.05; NS: nonsignificant.

**Table 4:** Mean values of thyroid function tests during different trimesters of pregnancy*.

Thyroid hormone	Nonpregnant women	Pregnant women		
		1st trimester	2nd trimester	3rd trimester
FT_4_ (pmol/L)	15.6^a^ ± 3.2	13.0^b^ ± 2.8	11.5^c^ ± 1.9	11.3^c,d^ ± 1.5
FT_3_ (pmol/L)	3.2^a^ ± 0.8	3.8^b^ ± 1.1	3.6^c^ ± 0.9	3.9^b,d^ ± 1.0
TSH (mIU/L)	1.4^a^ ± 0.6	1.2^b^ ± 1.1	1.7^c^ ± 1.1	2.3^d^ ± 1.3

*Different superscript letters (a, b, c, and d) indicate that the difference in mean values of thyroid tests in a row is statistically significant at *P* < 0.05.
